# Nomograms predict survival of patients with lymph node-positive, luminal a breast cancer

**DOI:** 10.1186/s12885-021-08642-6

**Published:** 2021-08-28

**Authors:** Yilun Li, Li Ma

**Affiliations:** The Fourth Hospital of Hebei Medical University, No. 169 Tianshan Street, Yuhua District, Shi Jiazhuang City, Hebei Province China

**Keywords:** Luminal A, Lymph node-positive, Nomograms, Prognosis

## Abstract

**Background:**

To develop nomograms for the prediction of the 1-, 3-, and 5-year overall survival (OS) and breast cancer-specific survival (BCSS) for patients with lymph node positive, luminal A breast cancer.

**Methods:**

Thirty-nine thousand fifty-one patients from The Surveillance, Epidemiology, and End Results (SEER) database were included in our study and were set into a training group (*n* = 19,526) and a validation group (n = 19,525). Univariate analysis and Cox proportional hazards analysis were used to select variables and set up nomogram models on the basis of the training group. Kaplan-Meier curves and the log-rank test were adopted in the survival analysis and curves plotting. C-index, calibration plots and ROC curves were used to performed internal and external validation on the training group and validation group.

**Results:**

Following independent factors were included in our nomograms: Age, marital status, grade, ethnic group, T stage, positive lymph nodes numbers, Metastasis, surgery, radiotherapy, chemotherapy. In both the training group and testing group, the calibration plots show that the actual and nomogram-predicted survival probabilities are consistent greatly. The C-index values of the nomograms in the training and validation cohorts were 0.782 and 0.806 for OS and 0.783 and 0.804 for BCSS, respectively. The ROC curves show that our nomograms have good discrimination.

**Conclusions:**

The nomograms may assist clinicians predict the 1-, 3-, and 5-year OS and BCSS of patients with lymph node positive, luminal A breast cancer.

## Background

Breast cancer is the most prevalent carcinoma in women. An estimated 268,000 American women were diagnosed with breast cancer in 2019, accounting for approximately 30% of all new cancer diagnoses in women, resulting in 41,760 deaths (15% of women’s cancer mortality) [1].

A variety of methods, including DNA sequencing and immunohistochemistry, have been used to study the mechanisms driving the occurrence and progression of breast cancer [2]. In order to facilitate identification and treatment of breast cancer with different characteristics, immunohistochemical markers are used to classify tumors into subtypes [3]. Hormone receptors (HRs), such as the estrogen receptor (ER) and the progesterone receptor (PR), and human epidermal growth factor receptor 2 (HER2) are important immunohistochemical markers. Four molecular subtypes are recognized immunologically based on these biomarkers: luminal A, luminal B, basal-like, and HER2 [4]. There is a correlation between the molecular subtypes and the prognosis of breast cancer [5]. Luminal A breast cancer is defined as breast cancers with the following expression characteristics: ER > 1%, PR ≥ 20%, HER2 negative, and Ki-67 < 14% [6]. Howlader et al. reported that the proportions of luminal A in the breast cancer subtypes was 72.7% [7]. Luminal A breast cancers have high hormone receptor expression, negative HER2 expression, and a low proliferation rate compared to other subtypes of breast cancer. Fortunately, these characteristics contribute to a better prognosis for patients with luminal A breast cancer [8, 9]. Yet, additional factors may affect the prognosis of this subtype.

Lymph node-positive is a high-risk factor of breast cancer and is related to lymph node metastasis. First, the expression of microRNA is an important factor affecting lymph node metastasis. Some studies have reported that the expression of miR-98 leads to metastasis of tumor cells to sentinel lymph nodes, which is associated with the poor prognosis of ER-positive, HER-2 negative breast cancer [10, 11]. Second, some immune cells are also associated with lymph node metastasis. A study by Takada et al. showed that the density of tumor-infiltrating lymphocytes in patients with lymph node metastasis was significantly lower than in patients without lymph node metastasis [12]. Third, the invasion of peripheral and lymphatic vessels is associated with lymph node metastasis. Çetintaş et al. reported that perineural invasion and lymphatic vessel invasion were significantly associated with the risk of lymph node metastasis [13]. In addition to the factors mentioned above, other factors such as tumor size, body mass index (BMI), and the platelet-to-lymphocyte ratio are also associated with lymph node metastasis and breast cancer prognosis [10, 14, 15].

Although it has been confirmed that some factors, such as body mass index (BMI) and the expression of the mircoRNAs mentioned above, affect the prognosis of lymph node positive, luminal A breast cancer, however, whether chemotherapy can improve the survival of these patients is still controversial. While the study by Herr et al. showed that the OS of patients with lymph node positive, luminal A breast cancer improved after receiving chemotherapy [16], studies by Taskaynatan H et al. and Uchida N et al. failed to show benefit from chemotherapy for the same patient population [17, 18]. Therefore, it is necessary to use chemotherapy as a predictor to build a predictive model, which can more accurately clarify and predict the impact of chemotherapy on the prognosis of patients with breast cancer. Moreover, a nomogram is a visual tool based on a prognostic model that includes relevant clinicopathological factors that provide specific individual clinical outcomes, thereby providing clinicians with a more accurate assessment of prognosis. Previous nomograms did not show the effect of the treatment on the survival of patients with luminal A, lymph node-positive breast cancer [19, 20]. but the treatment, for example, surgery, has a significant effect on the prognosis of breast cancer [21]. Thus, it is important to use the treatment modality as a predictor for building the nomogram to predict the prognosis of patients.

**Fig. 1:**
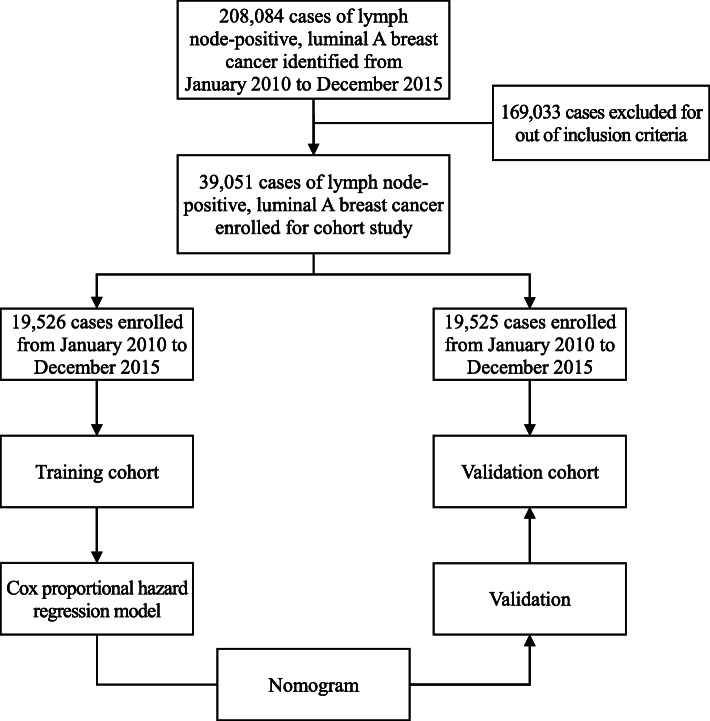
Flow diagram showing steps involved in construction and validation of nomograms

In this study, we focused on constructing nomograms which can predict the survival outcomes of patients with lymph node positive, luminal A breast cancer. First, the information of the patients was screened from the Surveillance, Epidemiology, and End Results (SEER) database. Then, the patients were divided into two groups, the test group and the verification group, and the test group was used to construct a model to predict the prognosis of the patients. Finally, the validation group was used to verify the sensitivity and accuracy of the model. Detailed information about the steps of construction and validation of the nomogram are presented in the Fig. 1.

## Methods

### Research populations

We collected and screened information from January 2010 to December 2015 in SEER Registry data of 18 registries. The following are the inclusion criteria: (1) Female; (2) Age of diagnosis ≥18; (3) Diagnosis confirmed by positive histology instead of other methods; (4) Breast cancer was considered as the first primary cancer; (5) The subtype of breast cancer is luminal A; (6) Complete survival data and survival time was not “0”; (7) Complete information of the variables contains age of diagnosis, ethnic group, marital status, historical subtype, tumor size, location, grade, laterality, positive lymph nodes counts, histological subtype, the seventh edition of American Joint Committee on Cancer (AJCC) TNM stage, tumor grade, SEER cause-specific death, vital status, breast cancer subtype and metastasis site (8) The TNM stage is T1–4, N1-N3 and M0-M1 according to the seventh edition of AJCC TNM.

### Variables and definition

The following data were extracted for each patient from the database: age at diagnosis, year diagnosed, race, marital status at diagnosis, primary site of the tumor, adjusted AJCC seventh T stage, N stage, M stage, tumor grade, histological subtype, number of positive lymph nodes, surgery, chemotherapy, radiotherapy, SEER cause-specific death, metastasis site, vital status, breast cancer subtype, and survival (months).

Histologic grades were classified into well differentiated (grade 1), moderately differentiated (grade 2), poorly differentiated (grade 3), and undifferentiated /anaplastic (grade 4). In terms of marital status, unmarried included single, divorced, separated, widowed, unmarried and family partner. In the racial classification, others include American Indian / Alaskan Aboriginal and Asian / Pacific Islander. We define Overall survival (OS) as the time from diagnosis to death, from any cause or until the last follow-up. Breast Cancer-specific survival (BCSS) was taken the definition of the time from diagnosis to death caused by breast cancer or to the last follow-up time. The endpoint of follow-up was December 2015.

### Data analysis

The amounts and percentages of each variable through summarizing were used to describe the basic characteristics of the groups. In the training group, we adopted univariate analysis and multivariate Cox regression analyses to determine the risk of each factor associated with prognosis of OS and BCSS, which were performed by SPSS software (IBM Corporation, USA, version 21). The factor was considered significant if *p* < 0.05. All significant factors in the univariate analysis were included in the multivariate Cox regression analyses. The significant variables in multivariate Cox regression analyses were selected for the final prognostic models in order to construct the nomograms. The final prognostic model was then used to predict the 1 -, 3 -, and 5-year outcomes of OS and BCSS. We validated the nomogram internally and externally both in the training group and in the validation group. Harrell Consistency Index (C-Index) and area under ROC curve (AUC) were used to evaluate the nomogram, with a higher C-index indicating a more accurate prognostic predictions [22]. The nomogram demonstrated good discriminative ability, with a C-index between 0.78 and 0.81. We also adopted the calibration plot to evaluate nomogram performance. The calibration plots along the 45-degree line indicate a perfect calibration model in which the predicted probabilities are identical to the actual outcomes [22]. The survival analysis and curve plotting was carried out using Kaplan-Meier curves and the log-rank test, respectively. We used SEER*Stat software (version 8.3.6; NCI, Bethesda, MD) to extract the data. The C-index, ROC curves, nomogram, calibration curves and Kaplan-Meier curves were generated in R with packages “rms”, “survival”, “foreign”, “timeROC” and “regplot” respectively.

## Results

### Demographics and Clinicopathological characteristics

A total of 39,051 cases were collected from the SEER database for this study (Fig. 1). The eligible patients were randomly divided into a training group (*n* = 19,526) and a validation group (*n* = 19,525) at the ratio of 1:1. Among all the patients, most patients were between 40 and 49 (20.5%), 50–59 (26.7%), and 60–69 (25.7%) years of age. As for the ethnic group, most of the patients were Caucasian (79.6%). In regard to histology classification, firstly, most of the patients presented with infiltrating duct carcinoma (72.8%). Secondly, about half of the patients presented with grade II oncology grades (53.0%). Thirdly, most patients were N1 stage (76.0%) and almost all patients were M0 stage (97.1%). Nearly all of the patients received surgery (99.0%) and most received radiotherapy (62.4%) and chemotherapy (64.1%). The rate of metastasis to the bone, brain, liver, lung was 2.0, 0.1, 0.4, and 0.5%, respectively. All variables displayed similar proportions in the validation group and the training group. Table 1 demonstrates the details of the baseline characteristics.

**Table 1:** Demographic and clinicopathologic characteristics of the patients

Characteristics	All patients (***n*** = 39,051)		Training cohort (***n*** = 19,526)		Validation cohort (***n*** = 19,525)	
	Number of patients	%	Number of patients	%	Number of patients	%
**Age**						
18–29	230	0.6	117	0.6	113	0.6
30–39	2205	5.6	1127	5.8	1078	5.5
40–49	8012	20.5	4001	20.5	4011	20.5
50–59	10,445	26.7	5208	26.7	5237	26.8
60–69	10,020	25.7	4982	25.5	5038	25.8
70–79	5646	14.5	2841	14.5	2805	14.4
≥ 80	2493	6.4	1250	6.4	1243	6.4
**Race**						
White	31,209	79.9	15,673	80.3	15,536	79.6
Black	4159	10.7	2028	10.4	2131	10.9
Other	3683	9.4	1825	9.3	1858	9.5
**Marital status**						
Married	23,133	59.2	11,620	59.5	11,513	59.0
Unmarried	15,918	40.8	7906	40.5	8012	41.0
**Grade**						
I	7140	18.3	3616	18.5	3524	18.0
II	20,702	53.0	10,331	52.9	10,371	53.1
III	11,133	28.5	5542	28.4	5591	28.6
IV	76	0.2	37	0.2	39	0.2
**T stage**						
T1	15,694	40.2	7883	40.4	7811	40.0
T2	17,685	45.3	8832	45.2	8853	45.3
T3	4342	11.1	2149	11.0	2193	11.2
T4	1330	3.4	662	3.4	668	3.4
**N stage**						
N1	29,668	76.0	14,855	76.1	14,813	75.9
N2	6248	16.0	3141	16.1	3107	15.9
N3	3135	8.0	1530	7.8	1605	8.2
**M stage**						
M0	37,936	97.1	18,971	97.2	18,965	97.1
M1	1115	2.9	555	2.8	560	2.9
**Histology**						
Infiltrating duct carcinoma	28,423	72.8	14,279	73.1	14,144	72.4
Lobular carcinoma	5024	12.9	2474	12.7	2550	13.1
Other	5604	14.4	2773	14.2	2831	14.5
**Location**						
Nipple	220	0.6	106	0.5	114	0.6
Central portion of breast	2944	7.5	1550	7.9	1394	7.1
Upper-inner quadrant of breast	3909	10.0	1965	10.1	1944	10.0
Lower-inner quadrant of breast	2018	5.2	1022	5.2	996	5.1
Upper-outer quadrant of breast	15,879	40.7	7868	40.3	8011	41.0
Lower-outer quadrant of breast	3671	9.4	1807	9.3	1864	9.5
Axillary tail of breast	238	0.6	121	0.6	117	0.6
Overlapping lesion of breast	10,172	26.0	5087	26.1	5085	26.0
**Laterality**						
Right: origin of primary	19,451	49.8	9765	50.0	9686	49.6
Left: origin of primary	19,600	50.2	9761	50.0	9839	50.4
**Tumor size, cm**						
≤ 1	3295	8.4	1624	8.3	1671	8.6
≤ 2	12,544	32.1	6320	32.4	6224	31.9
≤ 3	10,756	27.5	5358	27.4	5398	27.6
≤ 4	4943	12.7	2434	12.5	2509	12.9
≤ 5	2654	6.8	1367	7.0	1287	6.6
> 5	4859	12.4	2423	12.4	2436	12.5
**Positive regional nodes number**						
1–3	29,319	75.1	14,688	75.2	14,631	74.9
4–9	6769	17.3	3398	17.4	3371	17.3
≥ 10	2963	7.6	1440	7.4	1523	7.8
**Bone metastasis**						
No	38,272	98.0	19,147	98.1	19,125	98.0
Yes	779	2.0	379	1.9	400	2.0
**Brain metastasis**						
No	39,023	99.9	19,509	99.9	19,514	99.9
Yes	28	0.1	17	0.1	11	0.1
**Liver metastasis**						
No	38,888	99.6	19,436	99.5	19,452	99.6
Yes	163	0.4	90	0.5	73	0.4
**Lung metastasis**						
No	38,852	99.5	19,423	99.5	19,429	99.5
Yes	199	0.5	103	0.5	96	0.5
**Surgery**						
No	402	1.0	203	1.0	199	1.0
Yes	38,649	99.0	19,323	99.0	19,326	99.0
**Radiotherapy**						
No/Unknown	14,695	37.6	7356	37.7	7339	37.6
Yes	24,356	62.4	12,170	62.3	12,186	62.4
**Chemotherapy**						
No/Unknown	14,030	35.9	7069	36.2	6961	35.7
Yes	25,021	64.1	12,457	63.8	12,564	64.3

### Univariate and multivariate cox analysis and nomogram constructions

**Fig. 2:**
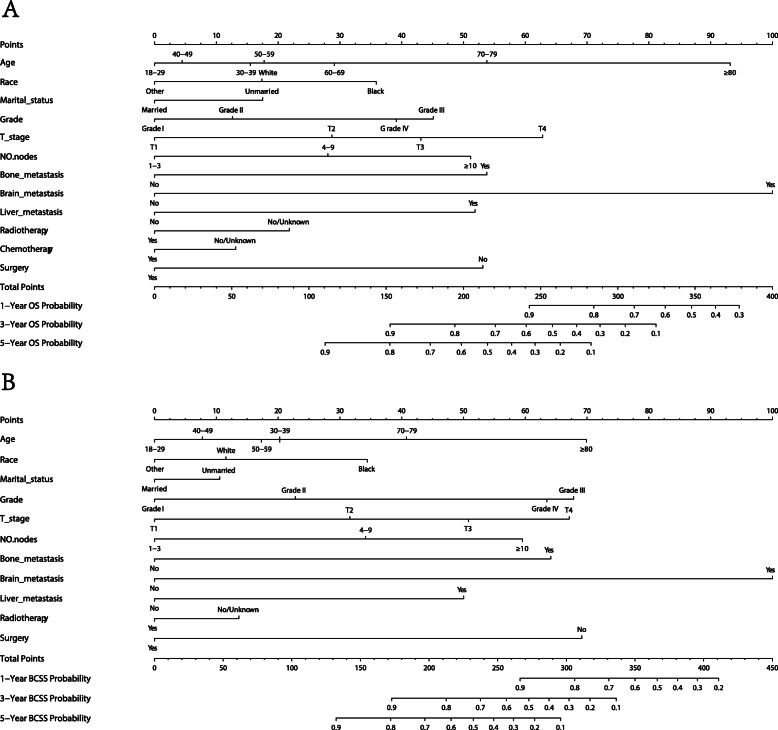
Nomograms for predicting 1-, 3-, and 5-year OS (**A**) and BCSS (**B**) for patients with the indicated prognosis factors. Summing up points from all predictors could obtain total points. The predicted probabilities of OS and BCSS can be obtained by projecting the location of the total points to the bottom scales. NO. nodes: number of positive lymph nodes; OS, overall survival; BCSS, breast cancer-specific survival

Univariate analyses showed that race, age of diagnosis, marital status, grade, T stage, Tumor size, N stage, M stage, positive regional nodes number, the site of metastasis (bone, brain, liver, lung), surgery records, radiotherapy records, chemotherapy records had a significant correlation with OS and BCSS (Table 2). According to the Cox regression multivariate analysis, the independent elements of OS and BCSS were identified and age of diagnosis, marital status, grade, T stage, M stage, race, positive regional nodes count, bone metastasis, brain metastasis, liver metastasis, surgery records, radiotherapy records, and chemotherapy records were independent prognostic factors. Black patients were observed to be at higher risk for death than Caucasian patients, while other patients have lower risk than Caucasian patients. The unmarried group was also found more to be at higher risk than the married group. In regard to histology classification, the risk of the Grade IV group was significantly higher than the Grade I group. The risk of the T4/N3/M1 group was obviously higher than the T1/N1/M0 group. With regard to treatment, patients who underwent surgery or received radiotherapy or chemotherapy were at lower risk than those who did not receive any of these treatments. As for breast cancer metastasis, patients with brain, bone, liver, and lung metastasis were at higher risk than those without. In the multivariate Cox proportional hazards models, we excluded M stage due to similar significance of the metastasis site and M stage while combining other independent predictors in the training group into the building of the nomogram for 1-, 3-, and 5-year OS and BCSS (Fig. 2). The length of the line behind the variable in the nomogram indicates the effect of the variable on the prognosis of breast cancer. From the nomogram, we found the brain metastasis, age, and T stage were the three most significant factors affecting the prognosis of patients with lymph node positive, luminal A breast cancer.

**Table 2:** Univariate and multivariate Cox analysis of overall survival and breast cancer-specific survival

Characteristics	Overall survival					Breast Cancer-specific survival				
	Log-rank test	Univariate analysis	***P*** value	Multivariate analysis	***P*** value	Log-rank test	Univariate analysis	*P* value	Multivariate analysis	***P*** value
	*P* value	HR (95% CI)		HR (95% CI)		*P* value	HR (95% CI)		HR (95% CI)	
**Age**	< 0.001					< 0.001				
18–29		Reference		Reference			Reference		Reference	
30–39		1.088 (0.550–2.154)	0.808	1.325 (0.668–2.628)	0.421		1.189 (0.578–2.446)	0.638	1.450 (0.702–2.993)	0.315
40–49		0.779 (0.401–1.515)	0.462	1.074 (0.551–2.092)	0.834		0.758 (0.374–1.535)	0.442	1.118 (0.551–2.271)	0.757
50–59		0.989 (0.511–1.914)	0.973	1.354 (0.698–2.626)	0.370		0.882 (0.437–1.778)	0.725	1.334 (0.660–2.699)	0.422
60–69		1.191 (0.616–2.303)	0.604	1.677 (0.865–3.250)	0.126		0.879 (0.435–1.772)	0.718	1.429 (0.706–2.892)	0.321
70–79		2.089 (1.080–4.041)	0.029	2.603 (1.341–5.053)	0.005		1.349 (0.667–2.726)	0.404	2.063 (1.016–4.190)	0.045
≥80		5.019 (2.594–9.712)	< 0.001	5.233 (2.685–10.200)	< 0.001		2.429 (1.197–4.929)	0.014	3.433 (1.673–7.043)	0.001
**Race**	< 0.001					< 0.001				
White		Reference		Reference			Reference		Reference	
Black		1.503 (1.333–1.694)	< 0.001	1.357 (1.200–1.535)	< 0.001		1.747 (1.517–2.012)	< 0.001	1.493 (1.291–1.726)	< 0.001
Other		0.661 (0.551–0.792)	< 0.001	0.732 (0.611–0.879)	0.001		0.799 (0.649–0.983)	0.034	0.807 (0.655–0.995)	0.045
**Marital status**	< 0.001					< 0.001				
Married		Reference		Reference			Reference		Reference	
Unmarried		2.012 (1.846–2.192)	< 0.001	1.362 (1.244–1.490)	< 0.001		1.671 (1.505–1.855)	< 0.001	1.212 (1.086–1.353)	0.001
**Grade**	< 0.001					< 0.001				
I		Reference		Reference			Reference		Reference	
II		1.426 (1.238–1.644)	< 0.001	1.249 (1.082–1.442)	0.002		1.873 (1.519–2.311)	< 0.001	1.540 (1.247–1.902)	< 0.001
III		2.742 (2.379–3.160)	< 0.001	2.204 (1.904–2.551)	< 0.001		5.043 (4.108–6.190)	< 0.001	3.557 (2.886–4.385)	< 0.001
IV		2.647 (1.248–5.612)	0.011	2.009 (0.945–4.274)	0.070		5.221 (2.293–11.889)	< 0.001	3.483 (1.523–7.965)	0.003
**T stage**	< 0.001					< 0.001				
T1		Reference		Reference			Reference		Reference	
T2		2.232 (2.002–2.489)	< 0.001	1.941 (1.121–3.362)	0.018		2.735 (2.365–3.163)	< 0.001	2.249 (1.209–4.183)	0.010
T3		3.315 (2.893–3.800)	< 0.001	1.737 (1.005–3.000)	0.048		4.910 (4.144–5.817)	< 0.001	2.479 (1.340–4.585)	0.004
T4		6.625 (5.621–7.808)	< 0.001	2.790 (1.681–4.632)	< 0.001		9.521 (7.791–11.635)	< 0.001	3.471 (1.962–6.143)	< 0.001
**N stage**	< 0.001					< 0.001				
N1		Reference		Reference			Reference		Reference	
N2		1.894 (1.706–2.103)	< 0.001	0.980 (0.757–1.268)	0.875		2.409 (2.122–2.734)	< 0.001	1.007 (0.751–1.350)	0.963
N3		3.234 (2.888–3.622)	< 0.001	1.029 (0.747–1.417)	0.862		4.570 (4.008–5.211)	< 0.001	1.124 (0.793–1.594)	0.511
**M stage**	< 0.001					< 0.001				
M0		Reference		Reference			Reference		Reference	
M1		5.668 (4.961–6.476)	< 0.001	1.779 (1.318–2.403)	< 0.001		8.160 (7.066–9.423)	< 0.001	2.191 (1.591–3.019)	< 0.001
**Histology**	0.223					0.233				
Infiltrating duct carcinoma										
Lobular carcinoma										
Other										
**Location**	0.012					0.353				
Nipple		Reference								
Central portion of breast		0.864 (0.519–1.437)	0.573							
Upper-inner quadrant of breast		0.659 (0.396–1.095)	0.108							
Lower-inner quadrant of breast		0.712 (0.422–1.202)	0.204							
Upper-outer quadrant of breast		0.653 (0.398–1.071)	0.091							
Lower-outer quadrant of breast		0.705 (0.423–1.173)	0.178							
Axillary tail of breast		0.925 (0.467–1.831)	0.823							
Overlapping lesion of breast		0.728 (0.443–1.196)	0.210							
**Laterality**	0.859					0.641				
Right: origin of primary										
Left: origin of primary										
**Tumor size, cm**	< 0.001					< 0.001				
≤1		Reference		Reference			Reference		Reference	
≤2		1.139 (0.904–1.435)	0.271	1.069 (0.848–1.348)	0.573		1.218 (0.883–1.680)	0.229	1.112 (0.805–1.537)	0.518
≤3		2.017 (1.611–2.526)	< 0.001	0.789 (0.448–1.390)	0.412		2.302 (1.686–3.144)	< 0.001	0.697 (0.365–1.329)	0.273
≤4		3.097 (2.456–3.906)	< 0.001	1.020 (0.580–1.794)	0.945		4.322 (3.153–5.926)	< 0.001	1.068 (0.561–2.034)	0.840
≤5		3.552 (2.782–4.534)	< 0.001	1.167 (0.661–2.058)	0.595		5.242 (3.782–7.266)	< 0.001	1.234 (0.648–2.352)	0.523
> 5		4.122 (3.283–5.176)	< 0.001	1.328 (0.765–2.306)	0.313		6.262 (4.595–8.534)	< 0.001	1.161 (0.621–2.171)	0.641
**Positive regional nodes number**	< 0.001					< 0.001				
1–3		Reference		Reference			Reference		Reference	
4–9		1.980 (1.789–2.192)	< 0.001	1.635 (1.270–2.104)	< 0.001		2.561 (2.265–2.896)	< 0.001	1.823 (1.367–2.430)	< 0.001
≥10		3.377 (3.010–3.788)	< 0.001	2.357 (1.698–3.270)	< 0.001		4.778 (4.179–5.463)	< 0.001	2.654 (1.852–3.802)	< 0.001
**Bone metastasis**	< 0.001					< 0.001				
No		Reference		Reference			Reference		Reference	
Yes		5.975 (5.130–6.959)	< 0.001	1.571 (1.159–2.129)	0.004		8.577 (7.289–10.093)	< 0.001	1.629 (1.179–2.251)	0.003
**Brain metastasis**	< 0.001					< 0.001				
No		Reference		Reference			Reference		Reference	
Yes		11.721 (6.479–21.204)	< 0.001	4.449 (2.381–8.313)	< 0.001		17.863 (9.865–32.342)	< 0.001	4.619 (2.440–8.745)	< 0.001
**Liver metastasis**	< 0.001					< 0.001				
No		Reference		Reference			Reference		Reference	
Yes		7.592 (5.630–10.238)	< 0.001	2.092 (1.494–2.929)	< 0.001		11.141 (8.191–15.155)	< 0.001	2.061 (1.457–2.916)	< 0.001
**Lung metastasis**	< 0.001					< 0.001				
No		Reference		Reference			Reference		Reference	
Yes		7.004 (5.303–9.249)	< 0.001	0.895 (0.644–1.243)	0.506		9.452 (7.020–12.7250	< 0.001	0.974 (0.686–1.382)	0.883
**Surgery**	< 0.001					< 0.001				
No		Reference		Reference			Reference		Reference	
Yes		0.220 (0.173–0.281)	< 0.001	0.401 (0.311–0.517)	< 0.001		0.169 (0.130–0.221)	< 0.001	0.288 (0.217–0.382)	< 0.001
**Radiotherapy**	< 0.001					< 0.001				
No/Unknown		Reference		Reference			Reference		Reference	
Yes		0.591 (0.543–0.643)	< 0.001	0.677 (0.619–0.741)	< 0.001		0.731 (0.658–0.812)	< 0.001	0.787 (0.704–0.881)	< 0.001
**Chemotherapy**	< 0.001					0.050				
No/Unknown		Reference		Reference			Reference		Reference	
Yes		0.577 (0.530–0.628)	< 0.001	0.792 (0.712–0.882)	< 0.001		0.897 (0.805–1.000)	0.051	0.937 (0.818–1.072)	0.340

### Validation of the nomograms

**Fig. 3:**
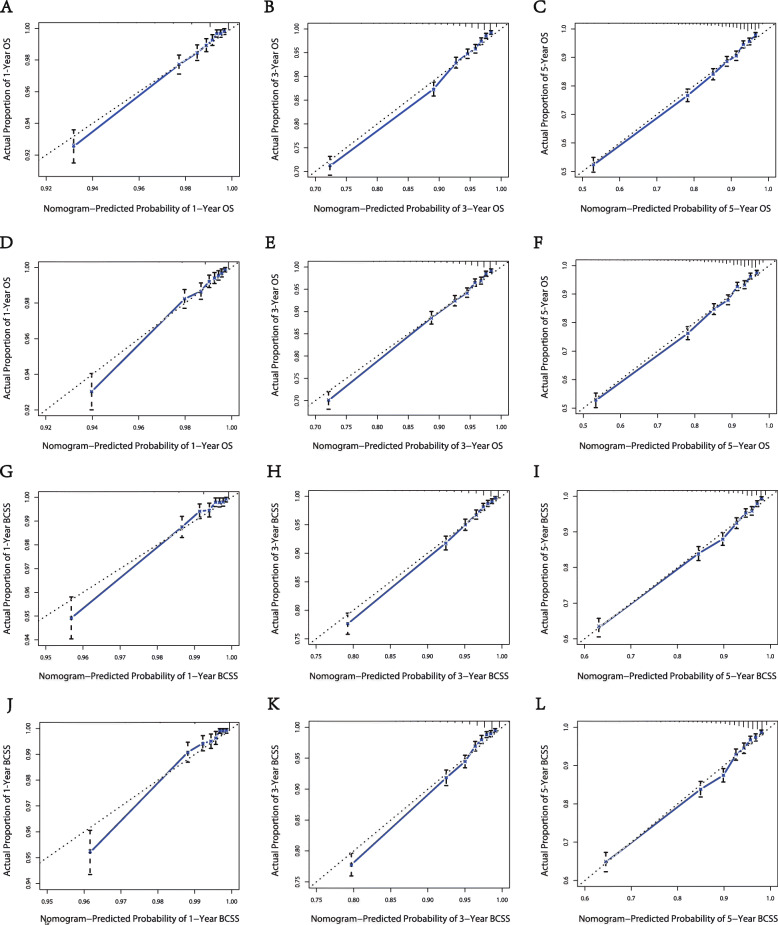
Calibration plots for the 1-, 3-, and 5-year. (**A**, **B**, **C**) Internal calibration curves for OS; (D,E,F) external calibration curves for OS; (**G**, **H**, **I**) internal calibration curves for BCSS; (**J**, **K**, **L**) external calibration curves for BCSS. OS, overall survival; BCSS, breast cancer-specific survival

**Fig. 4:**
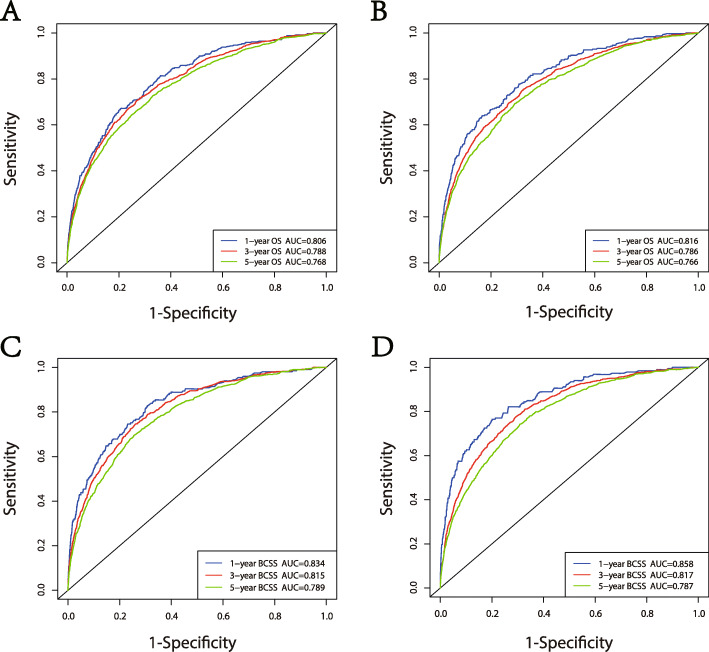
ROC curves for the 1-, 3-, and 5-year. (**A**) Internal calibration plots for OS; (**B**) external calibration curves for OS;(**C**) internal calibration plots for BCSS; (**D**) external calibration plots for BCSS. OS, overall survival; BCSS, breast cancer-specific survival

Our nomograms were validated internally and externally between the training group and the validation group. The calibration plots presented excellent consistency between the actual and nomogram-predicted survival probabilities in both the training the validation cohorts (Fig. 3). The AUC of the ROC curve, which indicates discrimination ability, in predicting 5-year OS was 0.768 in the training cohort and 0.766 in the validation cohort. The AUC of the ROC curve in predicting 5-year BCSS was 0.789 in the training cohort and 0.787 in the validation cohort (Fig. 4). Our findings indicate that the nomogram can efficiently predict a patient’s OS and BCSS.

Moreover, we determined the C-index values of our nomograms to assess their discriminative abilities. The C-index of OS were 0.782 (95% CI, 0.772–0.792) with 0.806 (95%CI, 0.794–0.818) for BCSS in the training cohort. In the testing cohort, C-index values for OS is 0.783 (95% CI, 0.773–0.793) and 0.804 (95% CI, 0.792–0.16) for BCSS.

### Survival analysis

**Fig. 5:**
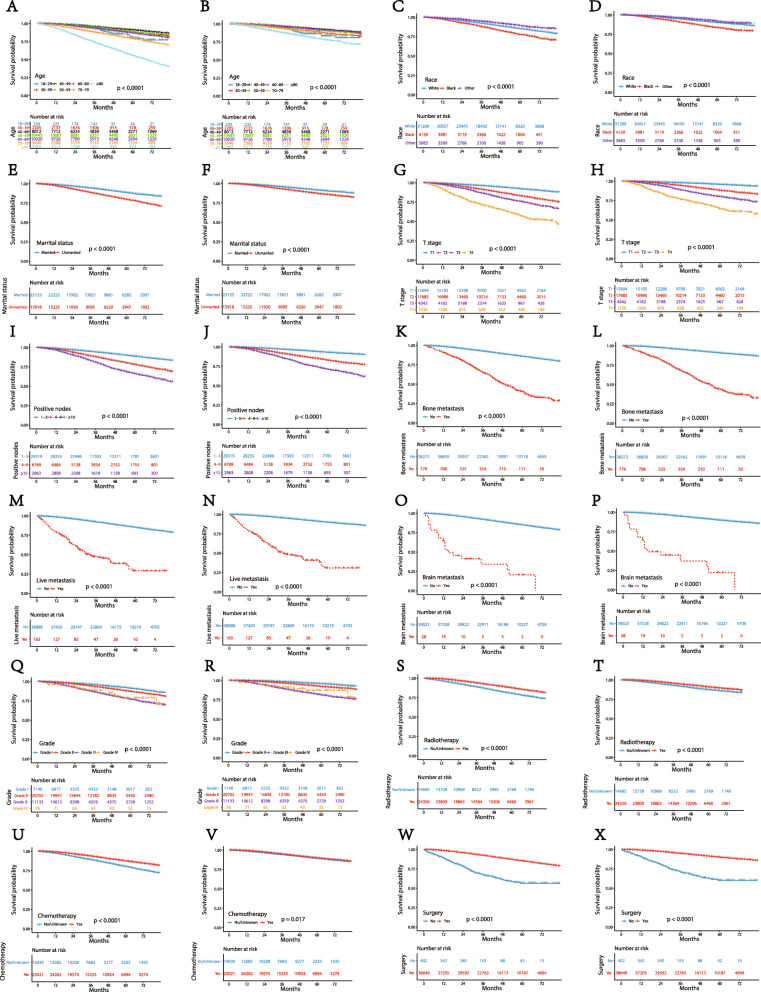
Kaplan-Meier curves of OS and BCSS for each predictor. (**A**, **B**) age; (**C**, **D**) race; (**E**, **F**) marital status; (**G**, **H**) T stage; (**I**, **J**) number of positive lymph nodes; (**K**, **L**) Bone metastasis; (**M**, **N**) liver metastasis; (**O**, **P**) brain metastasis; (**Q**, **R**) tumor grade; (**S**, **T**) Radiotherapy; (**U**, **V**) Chemotherapy; (**W**, **X**) Surgery

Kaplan-Meier curves were used to predict the effective factors of prognosis on the OS and BCSS of the test group in nomograms. The length of the line segment after the variable in the nomogram indicates the degree of influence of the variable on the prognosis of the patient. As shown in Fig. 2, brain metastasis has the most significant impact on the prognosis of patients. The Hazard Ratio of OS for patients with brain metastasis in the multivariate analysis was 4.449 (Table 2, 95% CI: 2.381–8.313). Surgery and the number of positive lymph nodes are also important factors affecting the prognosis of patients. The Hazard Ratio of OS for patients with surgery was 0.401 (95% CI: 0.311–0.517). The Hazard Ratio of OS patients with over 10 positive lymph nodes was 2.357(95%CI: 1.698–3.270). It is of great significance of all the prognostic factors in the nomograms in the primary group. Judging from Table 2, we observed consistent results in the training group. The curves indicates that all the factors turned out to have the identical outcome trends for OS and BCSS (Fig. 5).

## Discussion

Many factors are associated with the prognosis of lymph-node luminal A subtype breast cancer. So, it is vital to identify the independent factors related to prognosis. Nomograms were constructed to predict 1-, 3-, and 5-year OS and BCSS of patients, which contain the following risk elements: age of diagnosis, grade, ethnic group, T stage, marital status, positive regional nodes number, bone metastasis, brain metastasis, liver metastasis, surgery, radiotherapy, and chemotherapy.

One of the most significant risk factors that affects the prognosis of breast cancer was age at diagnosis. Liu et al. observed that patients with luminal A breast cancer had significantly lower 5-year disease-free survival (DFS) and distant metastasis-free survival (DMFS) in the ≤40 years old age group compared to the 41–60 years old age group [23]. Another study that young age at diagnosis is associated with lower frequency of luminal A breast cancer. The 5-year event-free survival rates of patients aged less than 40, between 40 and 50, and > 50 years were 54.3 ± 3.5, 68.5 ± 1.9, and 70.4 ± 1.3% [24]. Additionally, another study shows that breast cancer-specific mortality for age > 80 was 25.8% at 5 years [25]. In our study, the nomograms we constructed showed that, compared to the 40–49 age group, patients aged 18–29 at diagnosis have a lower risk of death, while patients aged 30–39 at diagnosis have a higher risk of death. When the age at diagnosis was ≥50, the risk of death generally showed a upward trend as age increased (Fig. 2). From Kaplan-Meier curves, we observed that the BCSS of the ≥80-year-old subgroup was not as bad as the OS (Fig. 5A, B). The study by *Chu* et al. also reported that age affects the prognosis, and their nomogram also shows that the ≥80-year-old subgroup had the highest risk compared to the other subgroups, which is consist with our results. Our findings suggest that the poor survival prognosis of patients aged ≥80 years old might be due to reasons other than the breast cancer itself. There are a number of reasons age may impact the prognosis of patients. First, the levels of estrogen and progesterone differ amongst patients in different age groups, and the levels of estrogen and progesterone are important factor that affect the occurrence and prognosis of breast cancer. Second, older patients are more likely to have chronic diseases, such as high blood pressure and diabetes. These diseases can also affect the survival of patients. Third, a study reported that older patients have a higher risk of venous thromboembolism after receiving chemotherapy or endocrine therapy [26].

Studies by Chu et al. and Wang et al. have observed that race is a factor related to the prognosis of breast cancer [19, 27]. From our nomograms, we observed American Indian/Alaska Native and Asian/Pacific Islander women have a lower risk of death compared to Caucasian women, while black women have a higher risk of death (Fig. 2). This is likely due to various reasons, such as medical conditions and environmental factors. A study reported that people often seek health care closer to them than at a greater distance [28]. The racial demographics differ in different areas and the incidence of medical conditions varies from region to region, which may be related to the stage at which the breast cancer is diagnosed and the conditions for the treatment of breast cancer. Being diagnosed at an advanced stage is often accompanied with poorer living conditions, ultimately affecting the prognosis of breast cancer [29].

The number of positive lymph nodes is one of the most important factors affecting the prognosis of patients with luminal A breast cancer. Studies by Han et al. and Herr et al. reported that the prognosis of patients with more than 3 positive lymph nodes was significantly worse than 1–3 positive lymph nodes in luminal A breast cancer [16, 30]. It also associated with distant recurrence. A study showed that patients with a ratio of ≤20% in the number of positive lymph nodes to the total number of excised auxiliary lymph nodes had lower distant recurrence and better OS than those with a ratio > 20% [31]. In our study, the hazard ratios of OS and BCSS shows an upward trend as the number of positive lymph nodes increases (Table 2) The number of positive lymph nodes is related to perineural invasion, lymphatic vessel invasion, and tumor size, all of which can affect the prognosis of luminal A breast cancer [13].

T stage, referring to the size of the tumor, also affects the prognosis of patients with luminal A subtype breast cancer. Kustic et al. study have reported larger tumor was related to poor prognosis and adversely affected DFS and OS [32]. Our study showed that the median survival time shortened as T stage increased (Fig. 5 G, H), which is consistent with the observations observed by Kustic et al. The nomogram constructed by Chu et al also shows an increased risk of death in the ≥5 cm group than that of the ≤1 cm group. The survival curve also shows that the survival time of the ≥5 cm group is noticeably shorter than that of the ≤1 cm group. This result may be due to the fact that larger tumors are often associated with later staging, and it is more likely to have lymph node metastasis and distant metastasis, which affects the prognosis of breast cancer [33].

The site of metastasis displays close correlation with the prognosis of breast cancer, and occurs in the bone, liver and brain of patients with luminal A breast cancer. Bone metastasis is the most common site of metastasis in luminal A breast cancer [34]. Parkes et al. reported that median survival time of bone-only metastasis is 7.54 years [35], while Wang et al. reported that the median survival time of liver metastasis was 15 months [36]. Brain metastasis is associated with poor prognosis of luminal A breast cancer. Kim et al. reported that the median survival of luminal A subtype in brain metastasis was 12 months, and it is 14 months for brain metastasis instead of visceral metastasis [37]. Our nomograms showed that patients with brain metastasis have the highest risk of death compared with liver and bone metastasis (Fig. 2). In our Kaplan-Meier curves, patients with bone metastasis had a similar median survival time as patients with liver metastasis in lymph node positive, luminal A breast cancer. Moreover, brain metastasis led to the shortest median survival time (Fig. 5 K-P). The reason why brain metastasis contributes to poor prognosis is that 80% of patients with breast cancer with brain metastases are accompanied by other extracranial diseases [38]. Additionally, patients with brain metastases are often at the later stage of the disease. These factors contribute the overall poor prognosis of patients with brain metastases.

The course of treatment also affects the prognosis of patients with lymph node positive, luminal A breast cancer. A study by Xue et al. reported that the OS of patients who underwent surgery was significantly longer than those treated without surgery (34 months versus 23 months, respectively) [39]. Surgery in auxiliary lymphadenectomy can improve the survival of breast cancer patients with lymph node positive breast cancer [21].

Radiotherapy is another important treatment option. A study shows that patients with luminal A breast cancer have the highest benefit of radiotherapy compared with other subtypes. This is due to the fact that luminal A breast cancers are radiosensitive, thus resulting in better response to the treatment, a reduced risk of recurrence, and increased survival [40].

Chemotherapy is another common treatment option for breast cancer patients. For patients with lymph node positive, luminal A breast cancer, The National Comprehensive Cancer Network (NCCN) guidelines recommend that patients receive chemotherapy regardless of the number of positive nodes [41]. Previous studies have also shown that patients with lymph node-positive, luminal A breast cancer can benefit from chemotherapy, which can prolong OS [42, 43]. However, not all patients with lymph node-positive, luminal A subtype breast cancer will benefit from chemotherapy. The National Surgical Adjuvant Breast and Bowel Project (NSABP) B20 and Southwest Oncology Group (SWOG) 8814 supposed that whether chemotherapy was needed was determined by the Oncotype DX 21-gene recurrence score (RS) [44, 45]. The SWOG 8814 study showed that postmenopausal women with lymph node-positive luminal A subtype breast cancer with low (< 18) or moderate (18 < RS < 31) recurrence scores do not benefit from chemotherapy [44]. In our work, surgery was considered to be the most important treatment option compared with chemotherapy and radiotherapy, according to the nomogram (Fig. 2). Patients who received radiotherapy, and surgery can prolong OS and BCSS according to Kaplan-Meier curves, but chemot, breaherapy did not improved BCSS as significantly as it improved OS (Fig. 5 S-X).

Other factors, such as breast feeding, marital status, and exposure to certain drug extracts, may also affect the prognosis of the patients with luminal A, lymph node-positive breast cancer. A previous study reported that breastfeeding may decrease the risk of breast cancer [46], and another study reported that breastfeeding may be related to the occurrence and prognosis of breast cancer [47]. With regard to marital status, a systematic review reported that unmarried women are more likely to develop advanced stage breast cancer, and that a spouse may represent an advantage for providing practical assistance and support that may lead to the early detection of the breast cancer [48]. Lastly, the hydroalcoholic extract of garden sage has been shown to inhibit the angiogenesis of breast cancer cell lines, thereby potentially improving the prognosis of patients with breast cancer [49].

Our study has established a prognostic model for patients with luminal A, lymph node-positive breast cancer, and our verification has shown that it has high accuracy and sensitivity. Besides, compared with previous similar studies, we have included the treatment method as a predictive factor, which could provide references for clinicians to choose appropriate treatment options for these patients. However, our study has several limitations. First, deviations due to race may exist in the study population due to the fact that most of the population in the SEER database is Caucasian. Therefore, whether our nomogram is applicable in other regions outside the United States of America needs to be investigated. Second, although internal and external validations were used to evaluate the performance of the nomograms, validating the nomograms in cohorts outside of the SEER program is still needed. Third, the SEER database lacks information about targeted therapy and endocrine therapy, so the effect of these treatments on the prognosis of patients with lymph node-positive, luminal A breast cancer could not be determined. Lastly, due to the lack of information in the SEER database, unknown information on chemotherapy and radiotherapy may affect the accuracy of our predictions. Therefore, further prospective studies are needed to guarantee the performance of our nomograms [21].

## Conclusion

Based on the information from the SEER database, nomograms were built to predict survival for lymph node positive, luminal A subtype of breast cancer. Compared with previous studies, this is the first nomogram that incorporates treatment as a predictor to predict the prognosis of luminal A, lymph node-positive breast cancer. Our validation analysis showed that the actual and nomogram-predicted survival probabilities were consistent and that our nomogram displays good discrimination. However, most of the population in the SEER database are Caucasian, and the lower proportion of blacks and Asians may affect the sensitivity and accuracy of the nomogram’s predictive qualities in these populations. The nomograms may provide clinicians with more information about the risky sides for each prognostic factor and may assist clinicians choosing the proper treatments that will increase the 1-, 3-, and 5-year OS and BCSS of patients with lymph node positive, luminal A breast cancer.

## Data Availability

The data generated and/or analyzed during the current study are available in the SEER public database (**https://seer.cancer.gov/**). All data is accessible through the SEER public database and we received administrative permission from the databases.

## Abbreviations

OS: Overall Survival
BCSS: Breast Cancer-Specific Survival
SEER: The Surveillance, Epidemiology, and End Results
HRs: Hormone receptors
ER: Estrogen Receptor
PR: Progesterone Receptor
HER2: Human Epidermal growth factor Receptor 2
BMI: Body Mass Index
AJCC: American Joint Committee on Cancer
ROC: Receiver Operating Characteristic
AUC: Area Under Curve
DFS: Disease-Free Survival
DMFS: Distant Metastasis-Free Survival
NCCN: National Comprehensive Cancer Network
NSABP: National Surgical Adjuvant Breast and Bowel Project
SWOG: Southwest Oncology Group
RS: Recurrence Score.
